# Outcomes with durvalumab after chemoradiotherapy in stage IIIA-N2 non-small-cell lung cancer: an exploratory analysis from the PACIFIC trial

**DOI:** 10.1016/j.esmoop.2022.100410

**Published:** 2022-03-02

**Authors:** S. Senan, M. Özgüroğlu, D. Daniel, A. Villegas, D. Vicente, S. Murakami, R. Hui, C. Faivre-Finn, L. Paz-Ares, Y.L. Wu, H. Mann, P.A. Dennis, S.J. Antonia

**Affiliations:** 1 Department of Radiation Oncology, Amsterdam University Medical Centers, Vrije Universiteit Amsterdam, Cancer Center Amsterdam, Amsterdam, The Netherlands; 2 Istanbul University—Cerrahpaşa, Cerrahpaşa School of Medicine, Istanbul, Turkey; 3 Tennessee Oncology, Chattanooga, USA; 4 Sarah Cannon Research Institute, Nashville, USA; 5 Cancer Specialists of North Florida, Jacksonville, USA; 6 Hospital Universitario Virgen Macarena, Seville, Spain; 7 Kanagawa Cancer Center, Yokohama, Japan; 8 Westmead Hospital and the University of Sydney, Sydney, Australia; 9 The University of Manchester and The Christie NHS Foundation Trust, Manchester, UK; 10 Universidad Complutense, CiberOnc, CNIO and Hospital Universitario 12 de Octubre, Madrid, Spain; 11 Guangdong Lung Cancer Institute, Guangdong Provincial People’s Hospital & Guangdong Academy of Medical Sciences, Guangzhou, China; 12 AstraZeneca, Cambridge, UK; 13 AstraZeneca, Gaithersburg, USA; 14 H. Lee Moffitt Cancer Center and Research Institute, Tampa, USA

**Keywords:** immunotherapy, radiation therapy, chemotherapy, surgery, multimodality therapy

## Abstract

**Background:**

The phase III PACIFIC trial (NCT02125461) established consolidation durvalumab as standard of care for patients with unresectable, stage III non-small-cell lung cancer (NSCLC) and no disease progression following chemoradiotherapy (CRT). In some cases, patients with stage IIIA-N2 NSCLC are considered operable, but the relative benefit of surgery is unclear. We report a post hoc, exploratory analysis of clinical outcomes in the PACIFIC trial, in patients with or without stage IIIA-N2 NSCLC.

**Materials and methods:**

Patients with unresectable, stage III NSCLC and no disease progression after ≥2 cycles of platinum-based, concurrent CRT were randomized 2 : 1 to receive durvalumab (10 mg/kg intravenously; once every 2 weeks for up to 12 months) or placebo, 1-42 days after CRT. The primary endpoints were progression-free survival (PFS; assessed by blinded independent central review according to RECIST version 1.1) and overall survival (OS). Treatment effects within subgroups were estimated by hazard ratios (HRs) from unstratified Cox proportional hazards models.

**Results:**

Of 713 randomized patients, 287 (40%) had stage IIIA-N2 disease. Baseline characteristics were similar between patients with and without stage IIIA-N2 NSCLC. With a median follow-up of 14.5 months (range: 0.2-29.9 months), PFS was improved with durvalumab versus placebo in both patients with [HR = 0.46; 95% confidence interval (CI), 0.33-0.65] and without (HR = 0.62; 95% CI 0.48-0.80) stage IIIA-N2 disease. Similarly, with a median follow-up of 25.2 months (range: 0.2-43.1 months), OS was improved with durvalumab versus placebo in patients with (HR = 0.56; 95% CI 0.39-0.79) or without (HR = 0.78; 95% CI 0.57-1.06) stage IIIA-N2 disease. Durvalumab had a manageable safety profile irrespective of stage IIIA-N2 status.

**Conclusions:**

Consistent with the intent-to-treat population, treatment benefits with durvalumab were confirmed in patients with stage IIIA-N2, unresectable NSCLC. Prospective studies are needed to determine the optimal treatment approach for patients who are deemed operable.

## Highlights

• The PACIFIC trial established durvalumab after CRT as standard of care for unresectable, stage III NSCLC.
• The optimum multimodal treatment strategy for patients with potentially resectable, stage IIIA-N2 NSCLC is unknown.
• Survival benefit with durvalumab was observed in patients with stage IIIA-N2, unresectable NSCLC in this post hoc analysis.
• Durvalumab after CRT also exhibited a manageable safety profile in this subpopulation from PACIFIC.
• Studies of surgical vs. non-surgical strategies are needed to establish the best approach for potentially operable patients.

## Introduction

The results of the phase III PACIFIC trial demonstrated that durvalumab monotherapy (for up to 12 months) improved survival in patients with unresectable, stage III non-small-cell lung cancer (NSCLC) and no disease progression following platinum-based, concurrent chemoradiotherapy (cCRT). Durvalumab [an anti-programmed cell death-ligand 1 (PD-L1) antibody] significantly improved progression-free survival [PFS: stratified hazard ratio (HR) = 0.52; 95% confidence interval (CI) 0.42-0.65; *P* < 0.0001] and overall survival (OS: stratified HR = 0.68; 95% CI 0.53-0.87; *P* = 0.00251) versus placebo in the intent-to-treat (ITT) population.^1^, ^2^, ^3^ Furthermore, durvalumab exhibited a manageable safety profile and did not detrimentally impact patient-reported outcomes compared with placebo.^2^, ^3^, ^4^ Based on these findings, the ‘PACIFIC regimen’ (consolidation durvalumab after platinum-based CRT) has been established as the new standard of care in this setting.

Stage IIIA-N2 NSCLC represents a heterogeneous disease group, with some patients being potentially eligible for thoracic surgery.^5^^,^^6^ Before the approval of durvalumab, the recommended therapeutic approach for patients with stage IIIA-N2 disease comprised multimodality treatment with chemotherapy, radiotherapy, and (in patients deemed operable) a surgical resection.^7^, ^8^, ^9^, ^10^ Given the clinically meaningful improvements demonstrated with the PACIFIC regimen across a wide population of patients with unresectable, stage III NSCLC, the potential utility of immunotherapy in this setting warrants further evaluation in patients with stage IIIA-N2 disease who are deemed operable. Here we report a post hoc, exploratory analysis of clinical outcomes with durvalumab in patients from the PACIFIC trial with or without stage IIIA-N2, unresectable NSCLC.

## Materials and methods

### Study design

The eligibility criteria and study design for PACIFIC (NCT02125461), a randomized, double-blind, international, multicenter trial, have been described previously.^2^^,^^3^ Briefly, eligible patients had histologically or cytologically documented stage III, unresectable NSCLC, (according to the International Association for the Study of Lung Cancer Staging Manual in Thoracic Oncology, version 7) and had not progressed after at least two cycles of platinum-based, cCRT. Enrolled patients were randomized in a 2 : 1 ratio to receive either durvalumab (10 mg/kg bodyweight; AstraZeneca, Gaithersburg, MD) or placebo intravenously once every 2 weeks for 12 months, or until confirmed disease progression, initiation of an alternative anticancer therapy, development of unacceptable toxicities, or withdrawal of consent. All patients provided written informed consent for participation in the trial, which was approved by relevant ethics committees and carried out in accordance with the International Conference on Harmonization Guidelines on Good Clinical Practice and the Declaration of Helsinki.

### Endpoints and assessments

The primary endpoints were PFS [by blinded independent central review as per Response Evaluation Criteria in Solid Tumors (RECIST) version 1.1] and OS. Secondary efficacy endpoints included time to death or distant metastasis (TTDM), objective response rate (ORR), and safety (graded as per Common Terminology Criteria for Adverse Events version 4.03).

A post hoc, exploratory analysis of these endpoints was conducted in subgroups comprising patients with or without stage IIIA-N2 NSCLC; the subgroup without stage IIIA-N2 disease included patients with all other stages [i.e. stage IIIA non-N2 (T3N1, T4N0, and T4N1) and stage IIIB].

### Statistical analyses

Analysis of RECIST-assessed endpoints (PFS, TTDM, and ORR) was based on the data cut-off (DCO) used for the primary analysis of PFS (13 February 2017). OS and safety were analyzed based on the DCO used for the primary analysis of OS (22 March 2018).

Treatment effects (HRs) for time-to-event endpoints were estimated using unstratified Cox proportional hazards models, with treatment being the only covariate. Medians and associated 95% CIs were estimated with the Kaplan–Meier method. Adverse events (AEs), all-cause pneumonitis (i.e. pneumonitis/radiation pneumonitis), and ORR were summarized descriptively. All data were summarized based on the ITT population except for safety data, which were summarized based on the as-treated population.

## Results

Of the 713 randomized patients, 287 (40.3%) had stage IIIA-N2 disease; 197/476 (41.4%) and 90/237 (38.0%) in the durvalumab and placebo arms, respectively. Baseline characteristics were broadly similar between patients with and without stage IIIA-N2 NSCLC (Table 1). Proportionally more patients with (versus those without) stage IIIA-N2 NSCLC were aged ≥65 years in both study arms and proportionally fewer received pre-CRT induction chemotherapy. Approximately one-quarter of patients were randomized to study treatment within 1-14 days of completing radiotherapy, regardless of stage IIIA-N2 status [67/287 (23.3%) patients with and 115/426 (27.0%) patients without stage IIIA-N2 NSCLC]. Within the placebo arm, proportionally more patients with (versus those without) stage IIIA-N2 NSCLC were male [67/90 (76.7%) versus 97/147 (66.0%)] and proportionally fewer had an objective tumor response during prior cCRT [34/90 (37.8%) versus 85/147 (57.8%)].

**Table 1:** Baseline demographics, disease characteristics, and prior anticancer therapy in patients with or without stage IIIA-N2 NSCLC

	Patients with stage IIIA-N2 NSCLC		Patients without stage IIIA-N2 NSCLC^a^	
	Durvalumab (*n* = 197)	Placebo (*n* = 90)	Durvalumab (*n* = 279)	Placebo (*n* = 147)
Median age (range), years	65.0 (33-83)	65.0 (23-90)	62.0 (31-84)	63.0 (40-89)
Age ≥65 years, *n* (%)	112 (56.9)	46 (51.1)	103 (37.0)	61 (41.5)
Male, *n* (%)	132 (67.0)	67 (76.7)	202 (72.4)	97 (66.0)
Race, *n* (%)				
White	140 (71.1)	59 (65.6)	197 (70.6)	98 (66.7)
Black or African American	4 (2.0)	1 (1.1)	8 (2.9)	1 (0.7)
Asian	50 (25.4)	29 (32.2)	70 (25.1)	43 (29.3)
Other/missing	3 (1.5)	1 (1.1)	4 (4.4)	5 (3.4)
Median weight (range), kg	70.0 (37-133)	69.0 (38-128)	68.5 (34-175)	67.0 (40-121)
WHO performance status, *n* (%)				
0	99 (50.3)	45 (50.0)	135 (48.4)	69 (46.9)
1	97 (49.2)	44 (48.9)	143 (51.3)	78 (53.1)
Missing	1 (0.5)	1 (1.1)	1 (0.4)	0 (0)
Histology				
Squamous	88 (44.7)	36 (40.0)	136 (48.7)	66 (44.9)
Non-squamous	109 (55.3)	54 (60.0)	143 (51.3)	81 (55.1)
AJCC disease stage				
IIIA	197 (100)	90 (100)	55 (19.7)	35 (23.8)
IIIB	0	0	212 (76.0)	107 (72.8)
Other	0	0	12 (4.3)	5 (3.4)
*EGFR/ALK* aberration status, *n* (%)				
Negative	130 (66.0)	63 (70.0)	187 (67.0)	102 (69.4)
Positive	12 (6.1)	7 (7.8)	17 (6.1)	7 (4.8)
Unknown^b^	55 (27.9)	20 (22.2)	75 (26.9)	38 (25.9)
PD-L1 expression level, *n* (%)				
<25%	79 (40.1)	43 (47.8)	108 (38.7)	62 (42.2)
≥25%	45 (22.8)	14 (15.6)	70 (25.1)	30 (20.4)
Unknown^b^	73 (37.1)	33 (36.7)	101 (36.2)	55 (37.4)
Prior radiotherapy (Gy), *n* (%)				
<54	0		3 (1.1)	0
≥54-≤66	180 (91.4)	86 (95.6)	262 (93.9)	131 (89.1)
>66-≤74	17 (8.6)	4 (4.4)	13 (4.7)	15 (10.2)
>74	0	0	0	0
Missing	0	0	1 (0.4)	1 (0.7)
Prior cytotoxic chemotherapy, *n* (%)				
Adjuvant	0	0	3 (1.1)	1 (0.7)
Induction	38 (19.3)	19 (21.1)	85 (30.5)	49 (33.3)
Definitive	197 (100)	90 (100)	278 (99.6)	146 (99.3)
Not applicable	0	0	1 (0.4)	0
Prior definitive CRT chemotherapy regimen, *n* (%)^c^	196 (99.5)^c^	90 (100)^c^	277 (99.3)^c^	146 (99.3)^c^
Cisplatin-based	103 (52.6)	51 (56.7)	163 (58.8)	78 (53.4)
Carboplatin-based	91 (46.4)	38 (42.2)	108 (39.0)	64 (43.8)
Cisplatin/carboplatin-based	2 (1.0)	1 (1.1)	6 (2.2)	4 (2.7)
Best response to prior therapy, *n* (%)^d^				
Complete response	3 (1.5)	1 (1.1)	6 (2.2)	6 (4.1)
Partial response	94 (47.7)	33 (36.7)	143 (51.3)	79 (53.7)
Stable disease	100 (50.8)	53 (58.9)	123 (44.1)	62 (42.2)
Progressive disease	0	0	2 (0.7)	0
Not evaluable/applicable	0	3 (3.3)	5 (1.8)	0
Time from radiotherapy end to randomization, *n* (%)				
<14 days	45 (22.8)	22 (24.4)	75 (26.9)	40 (27.2)
≥14 days	152 (77.2)	68 (75.6)	204 (73.1)	107 (72.8)

AJCC, American Joint Committee on Cancer; ALK, anaplastic lymphoma kinase; CRT, chemoradiotherapy; EGFR, epidermal growth factor receptor; Gy, units of gray; NSCLC, non-small-cell lung cancer; PD-L1, programmed cell death-ligand 1; WHO, World Health Organization.

a Patients with all other stages (including stages IIIA-N0/N1 and IIIB).

b No sample collected or no valid test result.

c Based on the definitive platinum-based chemotherapy regimen only.

d Based on the last cycle of therapy before entering the study.

Consistent with the ITT population, proportionally more patients completed the full 12-month treatment course in the durvalumab arm compared with the placebo arm, irrespective of stage IIIA-N2 status [stage IIIA-N2: 98/195 (50.3%) versus 27/89 (30.3%); all others: 134/278 (48.2%) versus 55/147 (37.4%)] (Supplementary Table S1, available at https://doi.org/10.1016/j.esmoop.2022.100410).

**Figure 1:**
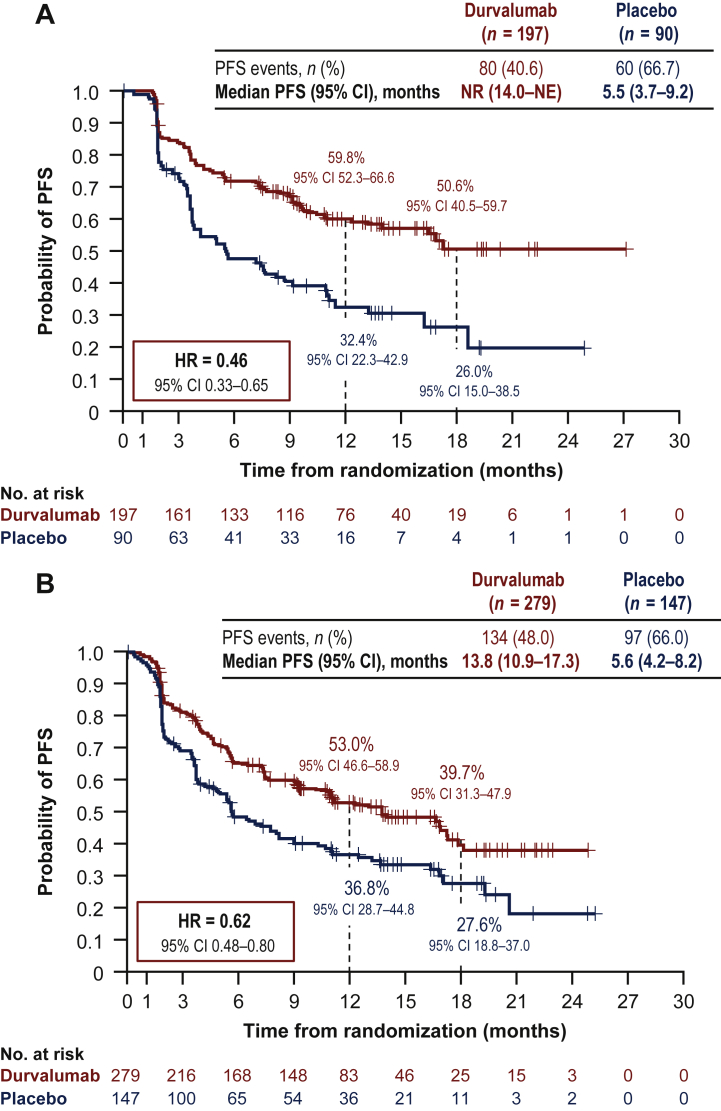
Shown are Kaplan-Meier curves for PFS. The tick marks indicate censored data, and the dashed vertical lines indicate the times of landmark analyses of PFS. PFS in patients (A) with or (B) without^a^ stage IIIA-N2 NSCLC. CI, confidence interval; DCO, data cut-off; HR, hazard ratio; NE, not estimable; NR, not reached; NSCLC, non-small-cell lung cancer; PFS, progression-free survival. ^a^Patients with all other stages (including stages IIIA-N0/N1 and IIIB). DCO = 13 February 2017 (DCO for the primary analysis of PFS): median follow-up of 14.5 months (range: 0.2-29.9 months).

**Figure 2:**
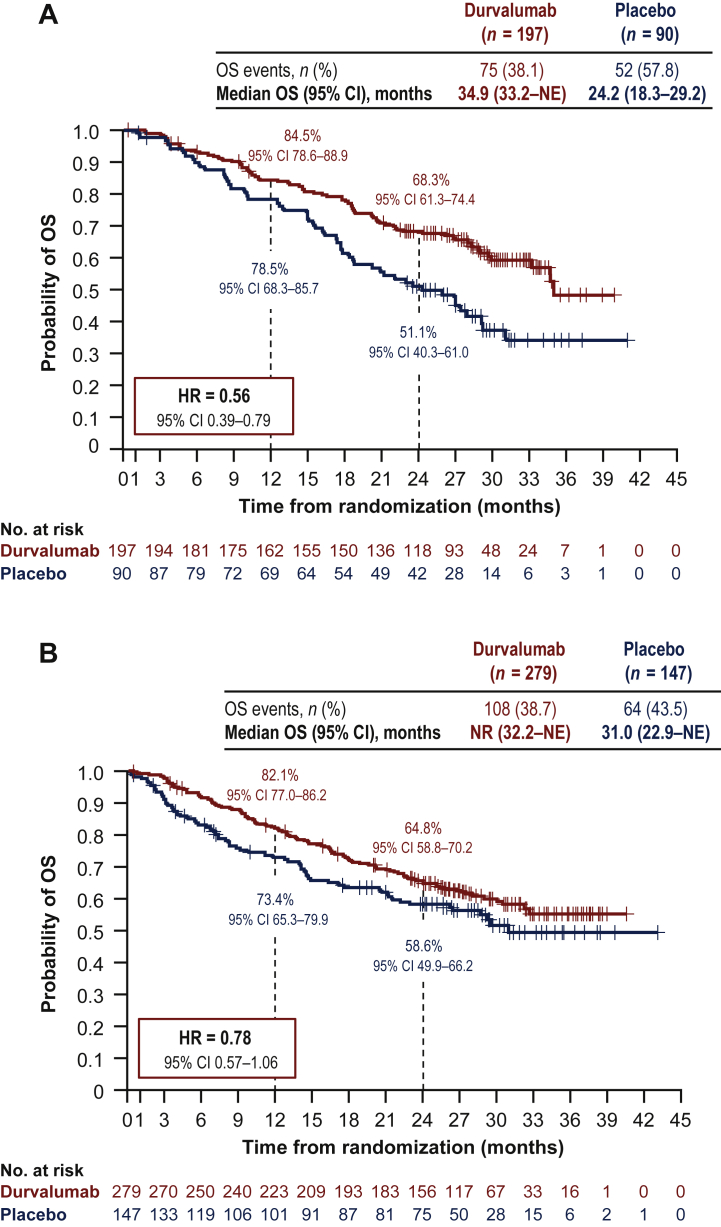
Shown are Kaplan-Meier curves for OS. The tick marks indicate censored data, and the dashed vertical lines indicate the times of landmark analyses of OS. OS in patients (A) with or (B) without^a^ stage IIIA-N2 NSCLC. CI, confidence interval; DCO, data cut-off; HR, hazard ratio; NE, not estimable; NR, not reached; NSCLC, non-small-cell lung cancer; OS, overall survival. ^a^Patients with all other stages (including stages IIIA-N0/N1 and IIIB). DCO = 22 March 2018 (DCO for the primary analysis of OS): median follow-up of 25.2 months (range: 0.2-43.1 months).

At the DCO for its primary analysis, PFS favored durvalumab compared with placebo in both patients with (HR = 0.46; 95% CI 0.33-0.65) and without (HR = 0.62; 95% CI 0.48-0.80) stage IIIA-N2 disease [Figure 1; DCO: 13 February 2017, median follow-up: 14.5 months (range: 0.2-29.9 months)]. Likewise, at the DCO for its primary analysis, OS favored durvalumab versus placebo in patients with (HR = 0.56; 95% CI 0.39-0.79) or without (HR = 0.78; 95% CI 0.57-1.06) stage IIIA-N2 disease [Figure 2; DCO: 22 March 2018, median follow-up: 25.2 months (range: 0.2-43.1 months)]. In addition, TTDM and ORR favored durvalumab versus placebo in both patients with and without stage IIIA-N2 disease (Table 2).

**Table 2:** TTDM and ORR in patients with or without stage IIIA-N2 NSCLC

	Patients with stage IIIA-N2 NSCLC		Patients without stage IIIA-N2 NSCLC^a^	
	Durvalumab (*n* = 197)	Placebo (*n* = 90)	Durvalumab (*n* = 279)	Placebo (*n* = 147)
TTDM events, *n* (%)	51 (25.9)	39 (43.3)	80 (28.7)	59 (40.1)
TTDM HR (95% CI)	0.45 (0.30-0.69)		0.63 (0.45-0.88)	
Median TTDM (95% CI), months	NR (NE-NE)	12.6 (8.7-25.9)	23.2 (17.8-NE)	14.8 (10.6-NE)
ORR, *n/N* (%)^b^	62/185 (33.5)	13/83 (15.7)	64/258 (24.8)	21/130 (16.2)
[95% CI]^c^	[26.8-40.8]	[8.6-25.3]	[19.7-30.5]	[10.3-23.6]

DCO = 13 February 2017 (DCO for the primary analysis of PFS): median follow-up of 14.5 months (range: 0.2-29.9 months). CI, confidence interval; DCO, data cut-off; HR, hazard ratio; NE, not estimable; NR, not reached; NSCLC, non-small-cell lung cancer; ORR, objective response rate; PFS, progression-free survival; TTDM, time to death or distant metastasis.

a Patients with all other stages (including stages IIIA-N0/N1 and IIIB).

b ORR for response-assessable patients includes unconfirmed responses (*N*; response-assessable population).

c ORR 95% CIs were calculated using the Clopper–Pearson method.

The incidences of any-grade, grade 3/4, and serious AEs were broadly similar between patients with and without stage IIIA-N2 disease irrespective of study treatment (Table 3). Consistent with the ITT population, a higher proportion of patients experienced any-grade pneumonitis/radiation pneumonitis with durvalumab versus placebo in both subgroups [stage IIIA-N2: 72/195 (36.9%) versus 24/89 (27.0%); all others: 89/280 (31.8%) versus 34/145 (23.4%)].

**Table 3:** Safety profile for patients with or without stage IIIA-N2 NSCLC

	Patients with stage IIIA-N2 NSCLC		Patients without stage IIIA-N2 NSCLC^a^	
	Durvalumab (*n* = 195)	Placebo (*n* = 89)	Durvalumab (*n* = 280)	Placebo (*n* = 145)
Any-grade all-causality AEs, *n* (%)	189 (96.9)	83 (93.3)	271 (96.8)	139 (95.9)
Grade 3/4	68 (34.9)	22 (24.7)	87 (31.1)	44 (30.3)
Outcome of death	9 (4.6)	5 (5.6)	12 (4.3)	10 (6.9)
Leading to discontinuation	32 (16.4)	8 (9.0)	41 (14.6)	15 (10.3)
Serious AEs, *n* (%)	59 (30.3)	19 (21.3)	79 (28.2)	35 (24.1)
Any-grade pneumonitis/radiation pneumonitis^b^, *n* (%)	72 (36.9)	24 (27.0)	89 (31.8)	34 (23.4)
Grade 1	21 (10.8)	11 (12.4)	46 (16.4)	14 (9.7)
Grade 2	39 (20.0)	7 (7.9)	33 (11.8)	15 (10.3)
Grade 3	10 (5.1)	3 (3.4)	7 (2.5)	3 (2.1)
Grade 4	0	0	0	0
Grade 5	2 (1.0)	3 (3.4)	3 (1.1)	2 (1.4)

DCO = 22 March 2018 (DCO for the primary analysis of OS): median follow-up of 25.2 months (range: 0.2-43.1 months). AE, adverse event; DCO, data cut-off; NSCLC, non-small-cell lung cancer; OS, overall survival.

a Patients with all other stages (including stages IIIA-N0/N1 and IIIB).

b Pneumonitis/radiation pneumonitis is a composite term that includes events of acute interstitial pneumonitis, interstitial lung disease, pneumonitis, pulmonary fibrosis, and radiation pneumonitis (alveolitis and diffuse alveolar damage were also included, but no events were found).

Within the durvalumab arm, there were limited differences in the receipt of post-discontinuation anticancer therapy between patients with and without stage IIIA-N2 disease (Supplementary Table S2, available at https://doi.org/10.1016/j.esmoop.2022.100410). However, within the placebo arm, proportionally more patients with (versus those without) stage IIIA-N2 disease received any post-discontinuation anticancer therapy [55/90 (61.1%) versus 73/147 (49.7%)]; in particular, post-discontinuation receipt of cytotoxic chemotherapy was higher among patients with stage IIIA-N2 disease, relative to those without.

## Discussion

In the PACIFIC trial, observed treatment benefits (in terms of PFS, OS, and TTDM) favored durvalumab versus placebo, with a manageable safety profile, irrespective of stage IIIA-N2 status. These findings were consistent with results for the ITT population.^1^, ^2^, ^3^

Stage IIIA-N2 NSCLC is a heterogeneous disease, with patients varying in terms of their fitness to undergo curative-intent treatment. Consequently, current treatment guidelines are somewhat complex, comprising multimodality regimens of chemotherapy with radiotherapy and/or surgical resection, with the combination and sequence of modalities tailored according to the disease characteristics of individual patients.^10^ As such, the optimal treatment sequence for this population has not been identified. Surgical treatment may be more appropriate in some patient subsets (e.g. those with minimal N2 disease) compared with others (e.g. those presenting with bulky or multilevel N2 disease).^8^^,^^11^ Importantly, there is, currently, no uniform definition of ‘resectable N2 disease’ and definitions can differ according to local practice and expertise.^6^ At present, the relative benefit of surgery has not been established definitively, with prospective randomized trials generally showing no significant differences in OS between patients with stage IIIA-N2 disease who did and who did not undergo surgical resection as part of a multimodality regimen.^12^, ^13^, ^14^, ^15^, ^16^, ^17^, ^18^ However, as much of the available data were generated before the current era of minimally invasive staging procedures/surgical resections and modern radiotherapy techniques (enabling improved tissue sparing),^19^^,^^20^ and as some key studies did not mandate histological confirmation of N2 disease, results of these older studies may not reflect the potential survival benefit in suitable subsets of patients with stage IIIA-N2 disease.

The findings of the present study (albeit carried out in patients with unresectable disease) emphasize the need to better define operability in the stage IIIA-N2 disease setting in order to demarcate patients who are more likely to benefit from surgery following chemotherapy/CRT. Future studies should seek to assess the impact of the PACIFIC regimen on progression patterns in patients with stage IIIA-N2 disease, as this information will help inform discussions regarding the optimum multimodal treatment approach for patients (given that surgery is expected to improve locoregional control). We did not analyze locoregional failure for the present study as radiotherapy planning parameters were not collected as part of the PACIFIC protocol. This is an important limitation of the study as it was not possible to correlate disease progression with prior radiation fields in all cases.

The treatment landscape for patients with resectable NSCLC is evolving rapidly. Recently, the US Food and Drug Administration approved use of adjuvant (anti-PD-L1) immunotherapy following resection and platinum-based chemotherapy for patients with stage II-IIIA NSCLC (and whose tumors have PD-L1 expression on ≥1% of tumor cells).^21^ In addition, several ongoing phase III trials are assessing neoadjuvant chemoimmunotherapy followed by surgery (e.g. NCT03800134, NCT02998528, and NCT03425643). The utility of immunotherapy in patients with stage IIIA-N2 NSCLC, including in patients who are deemed resectable (either in combination with or instead of surgery), remains unclear.^9^^,^^15^^,^^18^ Multiple early-phase clinical trials are evaluating neoadjuvant/adjuvant immunotherapy in patients with resectable, stage IIIA-N2 NSCLC.^22^^,^^23^ For example, data from a single-arm phase II study demonstrated a 1-year event-free survival rate of 73% (90% CI 63-82) with neoadjuvant/adjuvant durvalumab in this setting.^23^ However, larger comparative trials of surgical and non-surgical strategies for these patients are warranted.

The analyses reported in the present study are limited by their post hoc, exploratory nature. The PACIFIC trial was not designed to assess clinical outcomes with durvalumab according to specific disease stage, and complete details of mediastinal lymph node site involvement and gross tumor volumes were unavailable. In addition, details underlying the decision to deem each patient unresectable were not collected (with decision making at the discretion of the local principal investigator and associated multidisciplinary team), and study enrollment was not stratified according to TNM (tumor–node–metastasis) classification. Therefore, while clinical benefit with durvalumab (versus placebo) was observed irrespective of stage IIIA-N2 status, robust conclusions cannot be drawn due to the inherent lack of sufficient statistical power and imbalances in potentially prognostic baseline factors.

### Conclusions

In the PACIFIC trial, treatment benefit was observed with durvalumab versus placebo in patients with stage IIIA-N2, unresectable NSCLC. In the era of immune checkpoint inhibitors, prospective clinical studies comparing surgical and non-surgical strategies are warranted to establish the optimal treatment approach for patients with stage IIIA-N2 NSCLC who are potentially operable. Furthermore, improved and uniform definitions of patients who may or may not be suitable for surgery will be essential in this setting.
