# Supplementary material for: Outcomes with durvalumab after chemoradiotherapy in stage IIIA-N2 non-small-cell lung cancer: an exploratory analysis from the PACIFIC trial
Source: ESMO Open. 2022 Mar 2;7(2):100410. doi: 10.1016/j.esmoop.2022.100410 (PMC9058904; doi:10.1016/j.esmoop.2022.100410)
Supplement: Supplementary Tables S1 and S2 [file mmc1.docx]

**Supplementary Material**

**Supplementary Table S1. Patient disposition in patients with or without stage IIIA-N2 NSCLC**

|  | **Patients with  stage IIIA-N2 NSCLC** | | | **Patients without  stage IIIA-N2 NSCLC^a^** | | |
| --- | --- | --- | --- | --- | --- | --- |
|  | **Durvalumab**  **(*n* = 197)** | **Placebo**  **(*n* = 90)** | **Durvalumab**  **(*n* = 279)** | | **Placebo**  **(*n* = 147)** |  |
| **Received treatment, *n* (%)^b^** | 195 (99.0) | 89 (98.9) | 278 (99.6) | | 147 (100) |  |
| **Completed 12 months of treatment,**  ***n* (%)^c^** | 98 (50.3) | 27 (30.3) | 134 (48.2) | | 55 (37.4) |  |
| **Discontinued study treatment, *n* (%)^c^**  Subject decision  Adverse event  Severe non-compliance to protocol  Disease progression  Development of study specific discontinuation criteria  Other | 97 (49.7)  8 (4.1)  32 (16.4)  1 (0.5)  54 (27.7)  0  2 (1.0) | 62 (69.7)  4 (4.5)  8 (9.0)  0  50 (56.2)  0  0 | 144 (51.8)  6 (2.2)  41 (14.7)  0  94 (33.8)  1 (0.4)  2 (0.7) | | 92 (62.6)  8 (5.4)  15 (10.2)  1 (0.7)  67 (45.6)  1 (0.7)  0 |  |
| **Patients ongoing study at DCO, *n* (%)^b^** | 113 (57.4) | 36 (40.0) | 160 (57.3) | | 72 (49.0) |  |

DCO, data cut-off; NSCLC, non-small-cell lung cancer.

^a^ Includes patients with all other stages (including stages IIIA-N0/N1 and IIIB).

^b^ Denominator is all randomized patients.

^c^ Denominator is patients who received treatment only.

DCO = 22 March 2018 (DCO for the primary analysis of OS): median follow-up of 25.2 months (range: 0.2–43.1 months).

**Supplementary Table S2. Anticancer therapies received post-discontinuation of study treatment in patients with or without stage IIIA-N2 NSCLC**

|  | **Patients with  stage IIIA-N2 NSCLC** | | **Patients without  stage IIIA-N2 NSCLC^a^** | |
| --- | --- | --- | --- | --- |
|  | **Durvalumab**  **(*n* = 197)** | **Placebo**  **(*n* = 90)** | **Durvalumab**  **(*n* = 279)** | **Placebo**  **(*n* = 147)** |
| **Any, *n* (%)** | 71 (36.0) | 55 (61.1) | 124 (44.4) | 73 (49.7) |
| Radiotherapy | 30 (15.2) | 25 (27.8) | 52 (18.6) | 31 (21.1) |
| Immunotherapy^b^ | 17 (8.6) | 22 (24.4) | 21 (7.5) | 31 (21.1) |
| Cytotoxic chemotherapy | 44 (22.3) | 33 (36.7) | 84 (30.1) | 38 (25.9) |
| Other systemic therapies^c^ | 19 (9.6) | 12 (13.3) | 28 (10.0) | 19 (12.9) |
| Other | 1 (0.5) | 0 | 0 | 1 (0.7) |

DCO, data cut-off; NSCLC, non-small-cell lung cancer.

^a^ Includes patients with all other stages (including stages IIIA-N0/N1 and IIIB).

^b^ Primarily nivolumab or pembrolizumab.

^c^ Including tyrosine kinase inhibitors, among other treatments.

DCO = 22 March 2018 (DCO for the primary analysis of OS): median follow-up of 25.2 months (range: 0.2–43.1 months).
